# Embracing technological revolution: A panorama of machine learning in dentistry

**DOI:** 10.4317/medoral.26679

**Published:** 2024-10-13

**Authors:** Huili Lin, Jun Chen, Yinyi Hu, Wenjie Li

**Affiliations:** 1 Hunan Engineering Research Center for Oral Digital Intelligence and Personalized Medicine, Hunan Key Laboratory of Oral Health Research, Hunan Clinical Research Center of Oral Major Diseases and Oral Health, Academician Workstation for Oral-maxillofacial and Regenerative Medicine and Xiangya School of Stomatology, Central South University, Changsha, China; 2 Department of Periodontology, Xiangya Stomatological Hospital, Central South University, Changsha, China; 3 Department of Orthodontics, Xiangya Stomatological Hospital, Central South University, Changsha, China

## Abstract

**Background:**

The overarching aim of this study is to furnish dental experts and researchers with a comprehensive understanding of the role of machine learning in dentistry. This entails a nuanced understanding of prevailing technologies, discerning emerging trends, and providing strategic guidance for future research endeavors and practical implementations.

**Material and Methods:**

We assessed the literature by looking for papers related to the issue after 2019 in the Pubmed, Web of Science, and Google Scholar databases. A narrative review of 29 papers satisfying the search criteria was undertaken, with an emphasis on the application of machine learning in dentistry.

**Results:**

A review was conducted, including 29 publications. The advent of emerging technologies holds promise for enhancing the accuracy and efficiency of dental diagnosis, treatment, and prognosis. Nevertheless, the intricate nature of oral disease diagnosis and outcome prediction mandates acknowledgment of variables such as individual idiosyncrasies, lifestyle, genetics, image quality, and tooth morphology. These factors may impact the precision of machine learning models. Dental professionals should not rely solely on AI-based results but rather use them as references. Integrating these findings with clinical examinations, assessing the patient's overall health, and oral condition is crucial for informed decision-making.

**Conclusions:**

This review explores the clinical applications of machine learning in dentistry, encompassing disciplines like cariology, endodontics, periodontology, oral medicine, oral and maxillofacial surgery, prosthodontics and orthodontics. It serves as a valuable resource for dental practitioners and scholars in understanding the computer algorithms employed in each study, facilitating the clinical translation of machine learning research outcomes.

** Key words:**Machine learning, deep learning, artificial intelligence, dentistry, digital medicine.

## Introduction

Machine learning is a field of computer science that employs algorithms to identify patterns in data. Machine learning aims to enable computers to learn from data and enhance their performance without explicit programming, allowing them to make predictions, classifications, decisions, and perform other tasks. Basic machine learning algorithms can be broadly categorized into three types: supervised machine learning, unsupervised machine learning, and reinforcement machine learning, based on the tasks they are intended to solve (1). A comparison among three types of machine learning is shown in Table 1.

**Figure 1:**
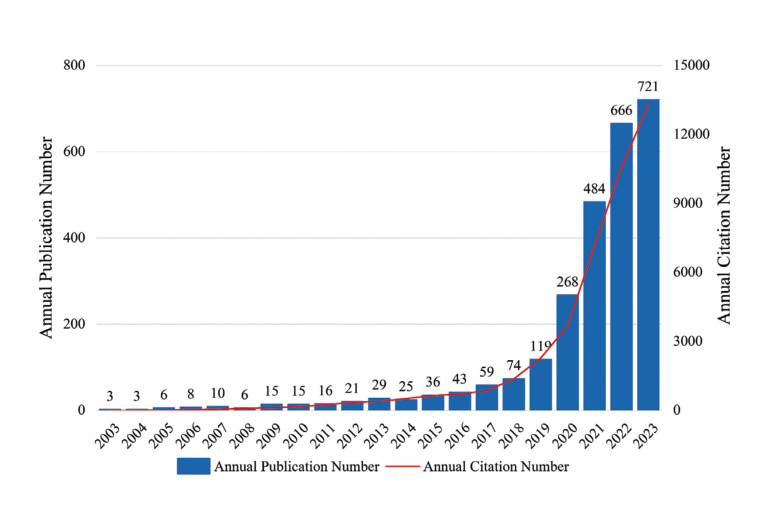
The annual publications and citations of machine learning in dentistry have shown a steady increase from 2004 to 2023.

**Figure 2:**
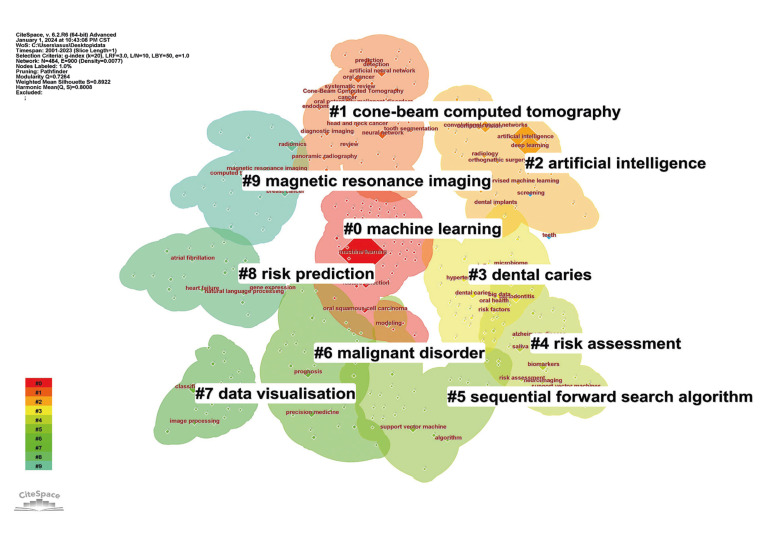
Co-occurrence clustering keyword in the field of machine learning in dentistry.

Machine learning has found widespread applications within the domain of dentistry (2). In recent years, the field has witnessed a substantial surge in applied research, reflected in a noTable increase in publications, as evidenced by Fig. 1. We analyzed the co-occurrence clustering of keywords in the field of machine learning and dentistry using CiteSpace(V6.2.R6). The top 5 most frequent keywords are “machine learning” (65 times), “cone beam computed tomography” (49 times), “artificial intelligence (AI)” (37 times), “dental caries” (36 times), “risk assessment” (35 times). The visualization graph shows these keywords, as delineated in Fig. 2.

From the outcomes of the two types of bibliometric analyses, it is discernible that AI in dental diagnostic imaging and risk assessment of oral diseases emerge as pivotal focal points for future research endeavors within the field of dentistry.

**Figure 3:**
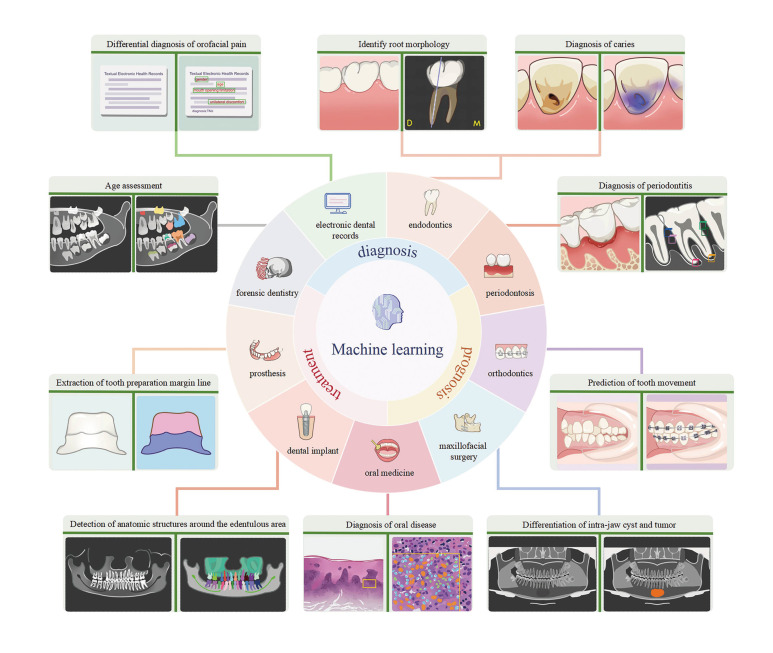
The application of machine learning in dentistry encompasses aiding in the diagnosis, prediction, and treatment planning of dental diseases.

A comprehensive summary of the applications of machine learning in dentistry is presented in Fig. 3. Through model training, machine learning facilitates the automatic identification and labeling of issues within patients' oral cavities, enabling dentists to identify and address potential oral health problems promptly and take preventive measures in advance to reduce the progression and severity of disease.

We provide a summary of the goals, machine learning algorithms, data sources, and limitations of the literature in this paper's Supplement 1. The overarching objective of this article is to furnish dental experts and researchers with a comprehensive understanding of the pivotal role that machine learning plays in dentistry. This contributes to a comprehensive understanding of current technologies, the identification of trends, and the provision of guidance for prospective research and pragmatic implementation.

## Material and Methods

In order to understand the application of machine learning in dentistry in recent years, a search was performed using Pubmed, Web of Science and Google Scholar databases, prioritizing literature published after 2019 for analysis. The terms "machine learning," "deep learning," "supervised machine learning," "support vector machine," and "unsupervised machine learning" were successively combined with the following terms: 1) caries: “Dental Decay” or “Dental Cavity” or "Carious Lesion" or "Lesion, Carious" or "Caries, Dental" or "Dental White Spot" or "Carious Dentin" or "Dentin, Carious", 2) Endodontics: "Endodontics" or "Endodontology" or "Dental Pulp Calcification" or "Dental Pulp Exposure" or "Dental Pulp Necrosis" or "Pulpitis" or "Root Canal Therapy" or "Root Canal Preparation", 3) Periodontal disease: “Periodontal Diseases” or “Periodontal” or “Parodontosis” or “Parodontoses” or “Pyorrhea Alveolaris” or “Furcation Defects” or “Gingival Diseases” or “Gingival Hemorrhage” or “Gingival Neoplasms” or “Gingival Overgrowth” or “Gingival Recession” or “Gingivitis” or “Alveolar Bone Loss” or “Periodontal Attachment Loss” or “Aggressive Periodontitis” or “Chronic Periodontitis” or “Periodontal Abscess” or “Periodontal Pocket”, 4) Oral Medicine: “oral mucosal disease”, 5) Orthodontic: “Orthodontic”, 6) Prosthodontics: “Prosthetic Dentistry” or “Dental Casting Technique" or "Dental Marginal Adaptation" or "Dental Prosthesis", and 7) Oral and Maxillofacial Surgery: "Oral Surgery" or "Maxillofacial Surgery". In addition to the manual search for the studies of machine learning in different oral diseases was conducted.

Inclusion criteria were (a) articles related to diagnostic, therapeutic, and prognostic studies of machine learning in dentistry, (b) assessment metrics including sensitivity, specificity, accuracy, precision, negative predictive value, area under the curve (AUC) or F-score (greater than or equal to 1), (c) full-text articles, and (d) published in English. Case reports and reviews were excluded.

Result

- Machine learning in caries

The utilization of visual examination is prevalent in caries detection. However, within clinical practice, divergent diagnoses from different dentists often arise. AI presents the potential for standardized diagnoses by analyzing intraoral photographs of teeth. Kühnisch *et al*. created a Convolutional Neural Network (CNN) -based deep learning algorithm to detect dental caries from intraoral photos. The algorithm achieved a 92.5% accuracy in caries detection and 93.3% accuracy with a cavity-related threshold (3). Zhu H *et al*. introduced an innovative deep learning architecture CariesNet to delineate various levels of dental caries in panoramic radiographs. The results demonstrated that the method’s effectiveness, achieving an average Dice coefficient of 93.64% and an accuracy of 93.61% in segmenting the three different levels of caries. However, CariesNet exhibited relatively lower performance in segmenting moderate caries (4). Lee S *et al*. developed a CNN model using U-Net for caries detection in bitewing radiographs. The algorithm showed high accuracy and consistent caries detection performance. It successfully identified various types of caries, except for proximal caries. However, there were challenges with low radiograph quality, dental overlap, and images including the third molar (5). A study developed machine learning models for predicting tooth decay in primary and permanent teeth after 2 and 10 years. The models considered clinical, socioeconomic, psychosocial, and behavioral factors, providing a comprehensive approach. Extreme Gradient Boosting (XGBoost) outperformed other models significantly in both follow-up periods (6).

- Machine learning in endodontics

Dental injury assessment: For dental vertical root fractures, Ziyang Hu *et al*. used the deep residual network (Resnet50) model to diagnose vertical root fracture (VRF) on Cone beam CT (CBCT) images, achieving an accuracy of 97.8% and sensitivity and specificity values of 97.0% and 98.5% respectively. Limitations include the need for more VRF data to enhance model stability, potential accuracy issues with non-endodontically treated teeth, artifacts from root filling materials and metal posts on CBCT images (7).

Evaluation of pulpal diseases: For the disease of pulp calcification, one study used deep learning to detect coronal pulp chamber and pulpal calcifications on bite-wing radiographs, employing the You Only Look Once version 4 (YOLOv4) algorithm. The achieved precision, specificity, and accuracy were 85.23%, 97.94%, and 96.54% (8). The multi-modal CNN, incorporating radiographs and clinical parameters using Resnet18, showed promise in accurately diagnosing deep caries and pulpitis with an accuracy of 0.86, precision of 0.85, sensitivity of 0.89, and specificity of 0.86. However, this study focused only on single carious lesions, and future research should explore the identification of multiple carious lesions (9).

Detection of periapical lesions: One study aimed to develop a deep learning method for accurately classifying and localizing radicular cysts and periapical granulomas in the mandible using panoramic imaging. The YOLOv3 network was used for lesion localization, resulting in high sensitivity and specificity for both cysts and granulomas. However, the study had limitations, including a small dataset, concerns about model interpretability, the need to balance specificity and sensitivity for clinical usability, and the impact of image quality on classification performance (10).

Assisting in root canal treatments: A deep learning model called EfficientNet was introduced to classify C-shaped canals of mandibular second molars using periapical and panoramic radiographs. The model achieved an AUC of 0.99, and its explainability was enhanced through Gradient-weighted Class Activation Mapping (Grad-CAM) and guided Grad-CAM, providing heat maps and highlighted outlines with high resolution (11).

- Machine learning in periodontal disease

Routine dental exams, the most efficient means of detection, are often unattainable in regions with limited medical resources or for low-income populations. In response to this challenge, Li W *et al*. developed a CNN with multi-task learning capabilities, trained on 3932 intraoral photos captured by smartphones. The model exhibited the ability to detect gingivitis, dental calculus, and dental debris (12). The researchers conducted a study using machine learning algorithms to differentiate between healthy individuals and those with varying degrees of periodontitis. The results consistently showed that certain pathogens played a significant role in distinguishing between healthy individuals and periodontitis patients. However, the study had limitations such as focusing only on common pathogens and not assessing host-derived salivary biomarkers. The small sample size from a single center also introduced potential bias (13). One study aimed to predict the 10-year survival of molars in patients with periodontal disease using machine learning models. The results showed that the ensembled model, combining neural network and logistic regression, performed the best. However, the study faced limitations due to variations in maintenance regimen and data collection methods among the different cohorts (14). A deep convolutional neural network was used to accurately measure the radiographic alveolar bone level and assist in periodontal diagnosis. The results demonstrated a strong agreement with human examiners in terms of segmentation and measurements. However, the model has limitations in accurately identifying the depth and angulation of vertical defects. It also struggles to accurately identify the position of missing teeth when multiple teeth are absent (15).

- Machine Learning in Oral Medicine

Diagnosis of oral mucosal diseases: A multilayer perceptron artificial neural network (ANN) was developed by Majdy Idreese *et al*. to detect cases of oral lichen planus (OLP) based on the count of inflammatory cells and mononuclear cells. The network accurately diagnosed OLP with a sensitivity of 100%, specificity of 91.25%, and accuracy of 94.62% by leveraging mononuclear cell count (16). In a different study, researchers used machine learning models to identify predictors of primary Sjögren's syndrome (pSS) and discovered potential therapeutic compounds for the disease. The Salmon module showed the strongest correlation with pSS and contained 239 genes. However, the study had limitations due to a small sample size and a lack of experimental data for the analyzed compounds (17).

Prediction of oral mucosal diseases: A pathomics-based model was developed to predict the malignant transformation risk of Oral Leukoplakia (OL) using H&E staining images from multi-center cohorts. Researchers combined resnet50 with LightGBM to craft a predictive pathomics-based model. However, further assessment in prospective clinical trials is required to gauge its predictive performance (18).

Treatment selection for oral mucosal diseases: Machine learning was used in a study on Burning Mouth Syndrome (BMS) to predict treatment outcomes based on patients' clinical data. The study employed Extreme Gradient Boosting Decision Trees methods and analyzed data from 420 primary type BMS patients. The accuracy of the models for initial treatment and clonazepam therapy predictions was 67.6% and 67.4%, respectively (19).

- Machine learning in orthodontic

Optimization of treatment plans: Several studies have already employed machine learning to assist in orthodontic diagnosis and treatment planning, particularly in identifying cephalometric landmarks (20) and the decision-making process for tooth extractions (21). A recent study developed a machine learning prediction model for forecasting orthodontic treatment plans. The study found that Decision Tree, Random Forest, and XGB classification algorithms achieved high accuracy, ranging from 87% to 93% (22).

Prediction of tooth movement: Abdul Rehman El Bsat *et al*. developed semantically individual teeth to create an autonomous system for assessing dentition from 2D images, validating the use of machine learning tools for precise tooth segmentation. However, the limitation of 2D imaging restricts the estimation of tooth motion to planar motion (x and y) and single rotation (z-axis) computed within the 2D image plane (23).

Evaluation of treatment effectiveness: A study utilized a conditional generative adversarial network (cGAN) to generate a 3D facial image post-orthodontic treatment. The cGAN, trained with paired CBCT datasets, considered factors such as gender, age, and incisor movement. The prediction error was estimated to be 1.2 ± 1.01 mm with an accuracy of 80.8% (24). Another study applied machine learning methods to evaluate the harmonization of craniodentofacial morphology after orthodontic treatment. The results indicated that the XGBoost regression model outperformed linear regression in terms of fitting and prediction. This study underscores the advantages of using the XGBoost regression model for evaluating craniodentofacial morphology, especially in scenarios with smaller sample sizes and limited transversal data (25).

Automated analysis: A study developed a web-based automated system using deep learning to address this issue. The system achieved accurate detection of cephalometric landmarks, with a point-to-point error of 1.37 ± 1.79 mm. Additionally, it successfully classified anatomical types with an accuracy rate of 88.43%. However, the algorithm's results may have been affected by the presence or absence of certain teeth in patient, introducing ambiguity (26).

- Machine learning in prosthodontics.

Automated analysis: In a study aiming to develop a CNN for accurately detecting posterior restorations in permanent teeth using intraoral clinical photographs, the CNN achieved a high diagnostic accuracy and specificity of over 90%, along with a sensitivity exceeding 80%. However, tooth-colored materials, specifically direct composite fillings, had the lowest diagnostic accuracy of 92.9% (27).Takahashi *et al*. found that dental arches can be classified using a CNN with high diagnostic accuracy. The prediction results were higher in edentulous cases without missing teeth due to a simple and uniform color distribution (28).

Prediction of treatment: Cui *et al*. developed a clinical decision support model using electronic dental records (EDRs) to predict the need for tooth extraction therapy. The study found that the XGboost models outperformed prosthodontists in terms of precision and conservatism. However, the model has limitations in only focusing on predicting oral conditions of teeth and struggles to consider multiple factors without affecting performance (29).

- Machine learning in Oral and Maxillofacial Surgery

Early diagnosis: A two-branch network was developed to classify jaw cysts and tumors using 872 lesion samples and 10,000 healthy samples, achieving an average accuracy of 88.72% (30). However, pathological samples are scarce compared to healthy samples during the machine learning training process. Fu *et al*. developed a deep learning algorithm to detect oral cavity squamous cell carcinoma (OCSCC) using photographic images. The algorithm achieved an accuracy of 92.3%, sensitivity of 91.0%, and specificity of 93.5% (31). However, the study did not include differential diagnosis with images of other oral diseases, such as oral and tuberculous ulcers.

Pathological evaluation: In oral oncological pathological evaluation, machine learning excels in analyzing pathological slide images to identify different types of tumor cells and tissue structures. A model utilizing a k-NN algorithm achieved an overall accuracy of 96.9% in distinguishing between oral epithelial dysplasia and oral squamous cell carcinoma (OSCC) (32). However, the study acknowledged the limitations of a relatively small dataset and the need for a more robust annotation process, impacting the models' accuracy and generalization ability.

Treatment decision-making: Machine learning-driven treatment recommendations improved survival rates for head and neck squamous cell carcinoma patients with intermediate risk factors (33). However, the inherent limitation of machine learning algorithms trained on large-scale population data raises the importance of considering individual patient variations for personalized treatment recommendations.

## Discussion

In recent years, machine learning has found applications across various disciplines in dentistry, ranging from caries, endodontics, periodontal disease, maxillofacial surgery, orthodontics, prosthodontics, and oral medicine. The use of machine learning algorithms as auxiliary tools for dentists holds great promise in ensuring top-notch dental treatments, with anticipated benefits including improved prediction of treatment outcomes, heightened diagnostic accuracy, and more effective treatment planning. Indeed, diagnosing and predicting treatment outcomes for oral diseases entail a complex task influenced by numerous factors, including individual differences, lifestyle, genetic factors, image quality, and tooth morphology. These variables may affect the accuracy of machine learning models. Dental professionals should not solely rely on AI-based detection results but rather use them as references. Combining these results with clinical examinations and assessments of the patient's overall health, and oral condition is vital for making informed and appropriate diagnostic and treatment decisions. While the application of machine learning algorithms in dental practice shows tremendous potential for the future, several challenges need to be addressed. Firstly, data privacy and security are crucial in dentistry (34), given the involvement of sensitive personal health data. Safeguarding this information may limit data sharing and access, making it challenging to train and optimize machine learning algorithms. Secondly, data quality is paramount for the performance of machine learning models. In dentistry, issues such as inconsistent, incomplete, or erroneous data may impact the algorithm's accuracy and generalizability (35). Another challenge is sample imbalance, where certain samples have lower representation in dental datasets, leading to decreased predictive performance for minority categories and difficulty obtaining accurate results (36). Additionally, the interpretability of machine learning algorithms is a key challenge (37). Dentists need to understand how models make diagnoses and treatment decisions, highlighting the importance of developing more interpreTable algorithms. Finally, the diversity of dental issues and patient variations poses a challenge to the generalization of machine learning models. In conclusion, while machine learning offers significant potential in dentistry, addressing these challenges is essential for its successful implementation.

## Conclusions

Machine learning is increasingly being applied in various clinical dental specialties, despite challenges in implementation. Advances in privacy-preserving techniques, data synthesis, interpretability methods, generalization capabilities, and multimodal fusion are expected to enhance its application in dentistry, elevating the level and quality of oral health services.

## Figures and Tables

**Table 1:** Comparisons among supervised machine learning, unsupervised machine learning, and reinforcement machine learning.

	Supervised Learning	Unsupervised Learning	Reinforcement Learning
Definition	Supervised learning is a machine learning paradigm where a model learns from labeled training data to make predictions or decisions on unseen data.	Unsupervised learning is a machine learning paradigm where a model learns patterns and structures in the data without explicit labels or guidance, aiming to discover hidden relationships or clusters within the data.	Reinforcement learning is a machine learning paradigm where an agent learns to make optimal decisions by interacting with an environment and receiving feedback in the form of rewards or penalties.
Training set	Possessing features and labels	No training set	No training set
Labels	Yes	No	No
Aim	In the face of data with only features and no labels, it is possible to infer the labels	Extracting features and structure from unlabeled data	Maximizing cumulative rewards or achieving specific goals through interaction with the environment
Interpretability	high	low	low
Classification	Classification Regression	Clustering Anomaly detection, Dimensionality reduction	Reinforcement Learning
